# Acquired urethral diverticulum in a man with paraplegia presenting with a scrotal mass: a case report

**DOI:** 10.1186/1752-1947-6-392

**Published:** 2012-11-21

**Authors:** Jalal Eddine El Ammari, Omar Riyach, Mustapha Ahsaini, Youness Ahallal, Mohammed Jamal El Fassi, My Hassan Farih

**Affiliations:** 1 Department of Urology, University Hospital Center Hassan II, New Fez 30000, Morocco; 2 Department of Urology, Hospital Center of Arras, Arras 62000, France

**Keywords:** Diverticulum, Paraplegic, Scrotal mass, Urethra

## Abstract

**Introduction:**

Male urethral diverticula are rare. Patients with paraplegia may present with acquired diverticula as a result of prolonged catheterization. Diverticula may be asymptomatic or lead to lower urinary tract symptoms. Rarely, the diverticulum may initially present as a scrotal mass.

**Case presentation:**

We report the case of a male 45-year-old Arab with paraplegia who presented with a mass in the peno-scrotal junction. He had in his medical history iterative prolonged urethral catheterizations associated with urine leakage through the urethral meatus upon applying compression. Diagnosis confirmation of urethral diverticula is obtained by retrograde urethrography. The patient underwent a diverticulectomy with urethroplasty.

**Conclusion:**

Male acquired urethral diverticula can be found in patients who have a spinal cord injury because of prolonged urethral catheterization. Clinical presentations are different and sometimes can be misleading. Retrograde urethrography is the key to diagnosis and open surgery is the treatment of reference.

## Introduction

Urethral diverticula are rare in males [1], and are always located in the penile urethra. They may be defined as pouch-like enlargements that are continuous with the urethral lumen. Acquired diverticula in males may be found anywhere along the urethra. The peno-scrotal junction is the most common site for anterior urethral diverticula. They can occur in patients who have a spinal cord injury as a result of repeated urethral trauma due to bladder catheterization [2,3].

## Case presentation

**Figure 1:**
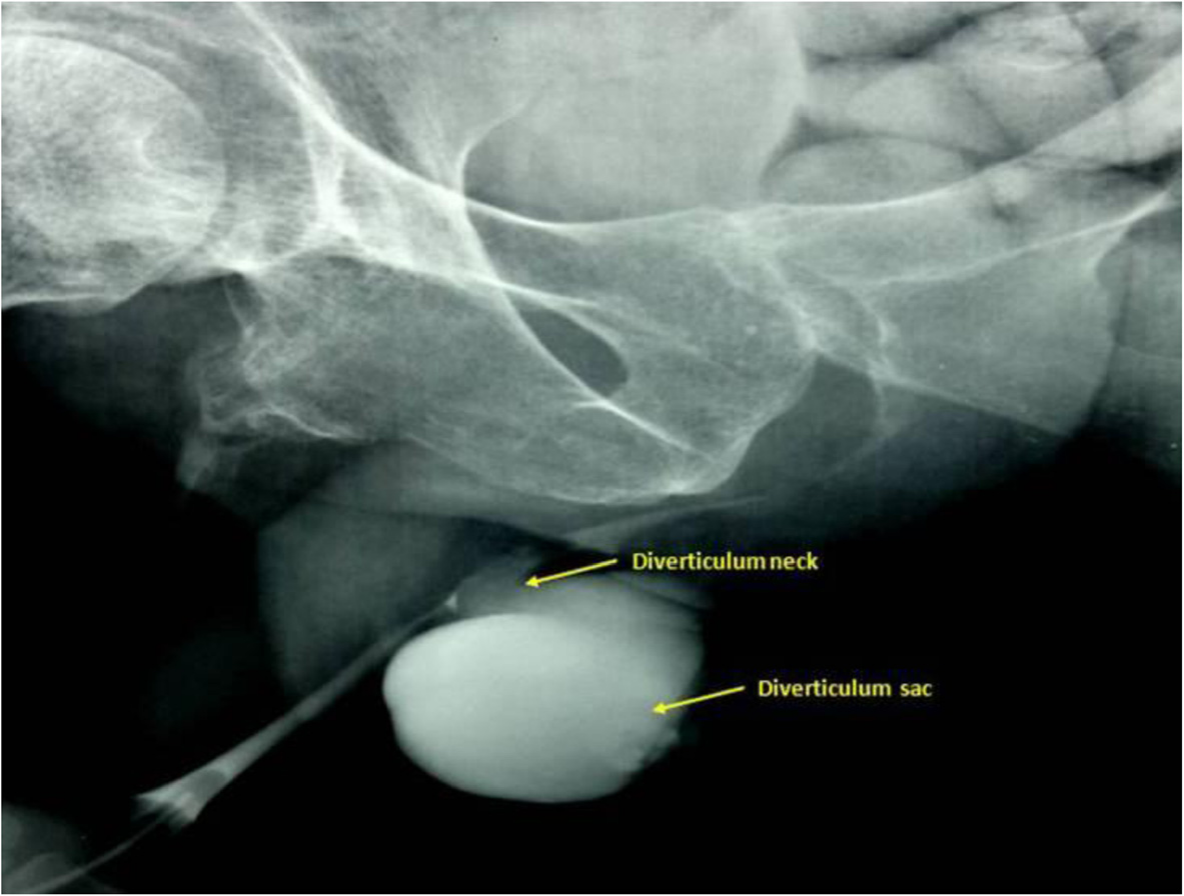
Retrograde urethrogram showing the urethral diverticulum and its neck (arrows) arising from the peno-scrotal urethra.

A male 45-year-old Arab presented with a three-cm mass in the peno-scrotal junction. He had paraplegia following a traffic accident 10 years earlier. In his medical history, the patient had presented with total urinary incontinence which was treated by iterative prolonged urethral catheterizations. He reported recurrent urinary infections and manual scrotal compression micturition for a number of months. On physical examination, there was a peno-scrotal junction mass mimicking a primary intrascrotal lesion with urine leakage through the urethral meatus upon applying compression. There was no bacterial or other growth on his urine culture. A retrograde urethrography revealed an anterior diverticulum filled with contrast (Figure 1).

**Figure 2:**
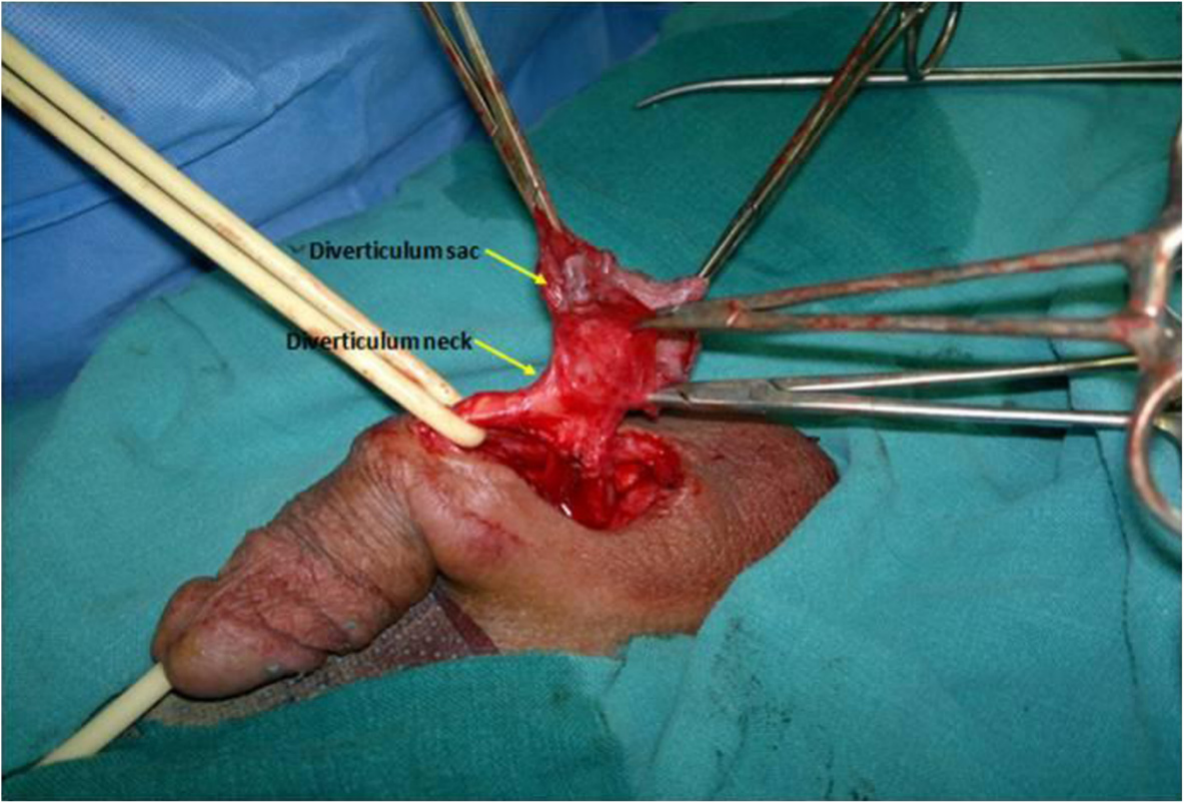
Complete exposition of the diverticulum and its neck (arrows) located in the peno-scrotal region.

**Figure 3:**
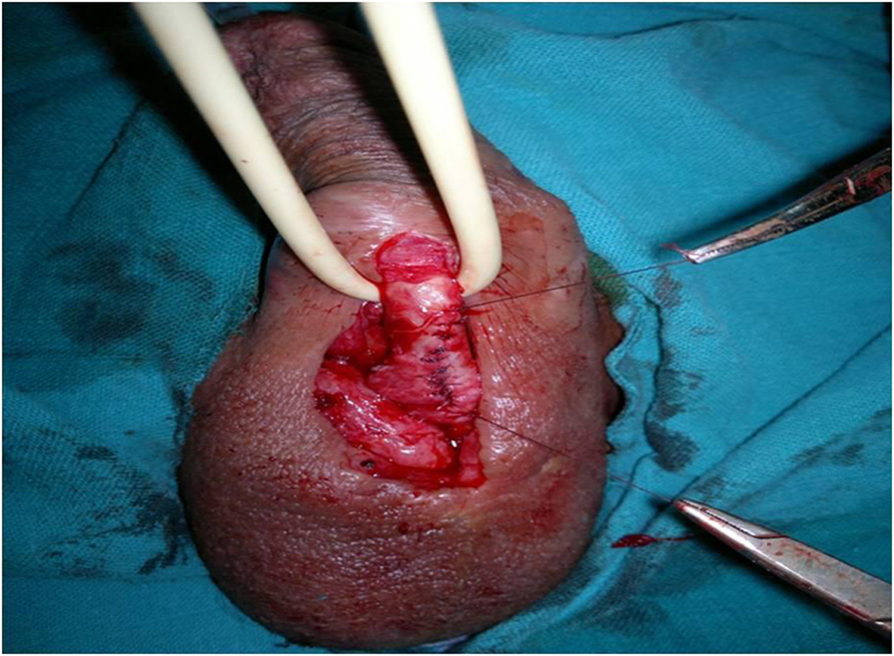
Urethroplasty performed following diverticulectomy.

The patient underwent open diverticulectomy and a primary urethroplasty. We first dissected the diverticulum and its entire neck until the diverticulum was completely exposed (Figure 2). The entire neck was removed, along with the urethral implantation zone, leaving a urethral defect of about one cm which was closed using 4–0 loose polydioxanone stitches (Figure 3). Penile dartos was interposed to avoid fistulas. Four weeks later, a retrograde urethrography showed a normal urethra, and the same result was recorded after one year of follow-up.

## Discussion

Male urethral diverticula are rare, and most (80% to 90%) are acquired [1]. They may be caused by blockage of the periurethral glands into the urethral lumen (cavity) with epithelialization (regrowth of tissue) over the opening of the resulting periurethral cavity. In a man with paraplegia, as in our case, long-term or clean intermittent catheterization to eliminate urinary stasis (neurogenic bladder) may induce, in the long term, repeated trauma and infection injuring the urethral wall and causing stricture, fistula and, to a lesser extent, diverticulum [2,3]. In some cases, diverticula may also appear as a complication of an artificial urinary sphincter, and after urethral surgery. Diagnosis of urethral diverticulum may be delayed because of the non-specific lower urinary tract symptoms. Patients may complain of recurrent urinary tract infections, pelvic pain, incontinence, post-void dribbling, dysuria (burning or pain with urination), urinary frequency and urgency, nocturia, or feeling of incomplete bladder emptying [4]. The diverticulum could present as a perineo-scrotal mass if the size is extremely large. In differential diagnosis of a scrotal mass in a male with paraplegia, especially when a urinary tract infection is observed, epididymo-orchitis and urethro-scrotal abscess should also be suspected. Retrograde urethrography is the best diagnostic technique to confirm and characterize the diverticulum [5]. However, Goyal *et al*. [6] have used transrectal sonography to diagnose congenital urethral diverticulum, but there is no recommendation of its routine use for acquired urethral diverticulum. Magnetic resonance imaging (MRI) is also useful because it can inform of the extent of the diverticulum, and of the involvement or not of the adjacent spongy tissue.

The most commonly reported associated complications are urinary tract infection, fistulas and stone formation in the diverticulum. Even adenocarcinoma has been reported to arise as a complication from urethral diverticulum.

On histopathological evaluation, acquired diverticula are generally lined by granulation tissue whereas congenital diverticula are lined by epithelia [6,7].

The recommended treatment is surgical excision of the diverticulum and urethroplasty over a transurethral catheter. If the urethral defect is very large, then extragenital free grafts can be used. In some cases an endoscopic approach has been applied to small diverticula. After surgical treatment, there is no perioperative morbidity, and the long-term follow-up results can be marked by the recurrence of diverticulum, fistula formation and urethral stenosis [8]. The follow-up of our case report was uneventful after one year.

## Conclusion

Diagnosing and treating urethral diverticula are very challenging. Peno-scrotal urethral diverticula may clinically mimic primary intrascrotal mass lesions. In clinical suspicion, retrograde urethrography is required as a gold standard method in the diagnosis of a urethral diverticulum. Open diverticulectomy and urethroplasty is the recommended approach to deal with large diverticula.

## Consent

Written informed consent was obtained from the patient for publication of this manuscript and accompanying images. A copy of the written consent is available for review by the Editor-in-Chief of this journal.

## Competing interests

The authors declare that they have no competing interests.

## Authors’ contributions

JEA, OR and MA the principal authors, were the major contributors in writing the manuscript. YA, MJE and MHF analyzed and interpreted the patient’s data and reviews of the literature. All authors read and approved the final manuscript.
